# Supplementary material for: Silver-Nanoparticles Embedded Pyridine-Cholesterol Xerogels as Highly Efficient Catalysts for 4-nitrophenol Reduction
Source: Materials (Basel). 2020 Mar 25;13(7):1486. doi: 10.3390/ma13071486 (PMC7177945; doi:10.3390/ma13071486)
Supplement: Supplementary file 1 [file materials-13-01486-s001.pdf]

materials

Supplementary Materials: Silver-nanoparticles Embedded Pyridine-Cholesterol Xerogels as Highly Efficient Catalysts for 4-nitrophenol Reduction

Ganesh Shimoga *, Eun-Jae Shin and Sang-Youn Kim *

Interaction Laboratory of Advanced Technology Research Center, Korea University of Technology and Education, Cheonan-si, Chungcheongnam-do, 330-708, South Korea.

* Correspondence: shimoga@koreatech.ac.kr (G.S.); sykim@koreatech.ac.kr (S.-Y.K.); Tel.: +82-(0)41-560-1484 (S.-Y.K.)

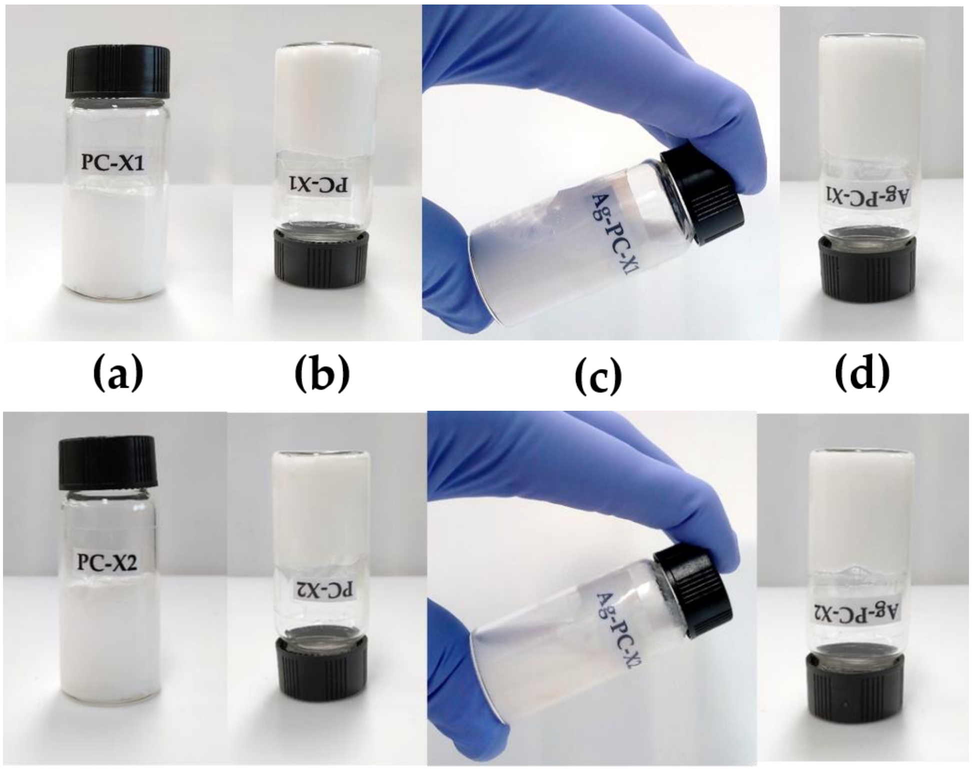

Figure S1. Pictograph representing the xerogel formation, (**a**) PC-X1 and PC-X2 after 15h, (**b**) Inverted PC-X1 and PC-X2 gels after 15h, (**c**) Ag-PC-X1 and Ag-PC-X2 in 0.05 M $AgNO_3$ solution in ethanol and acetone respectively after 5 min, (**d**) Inverted Ag-PC-X1 and Ag-PC-X2 gels after 15h.

Energy-dispersive X-ray spectroscopy

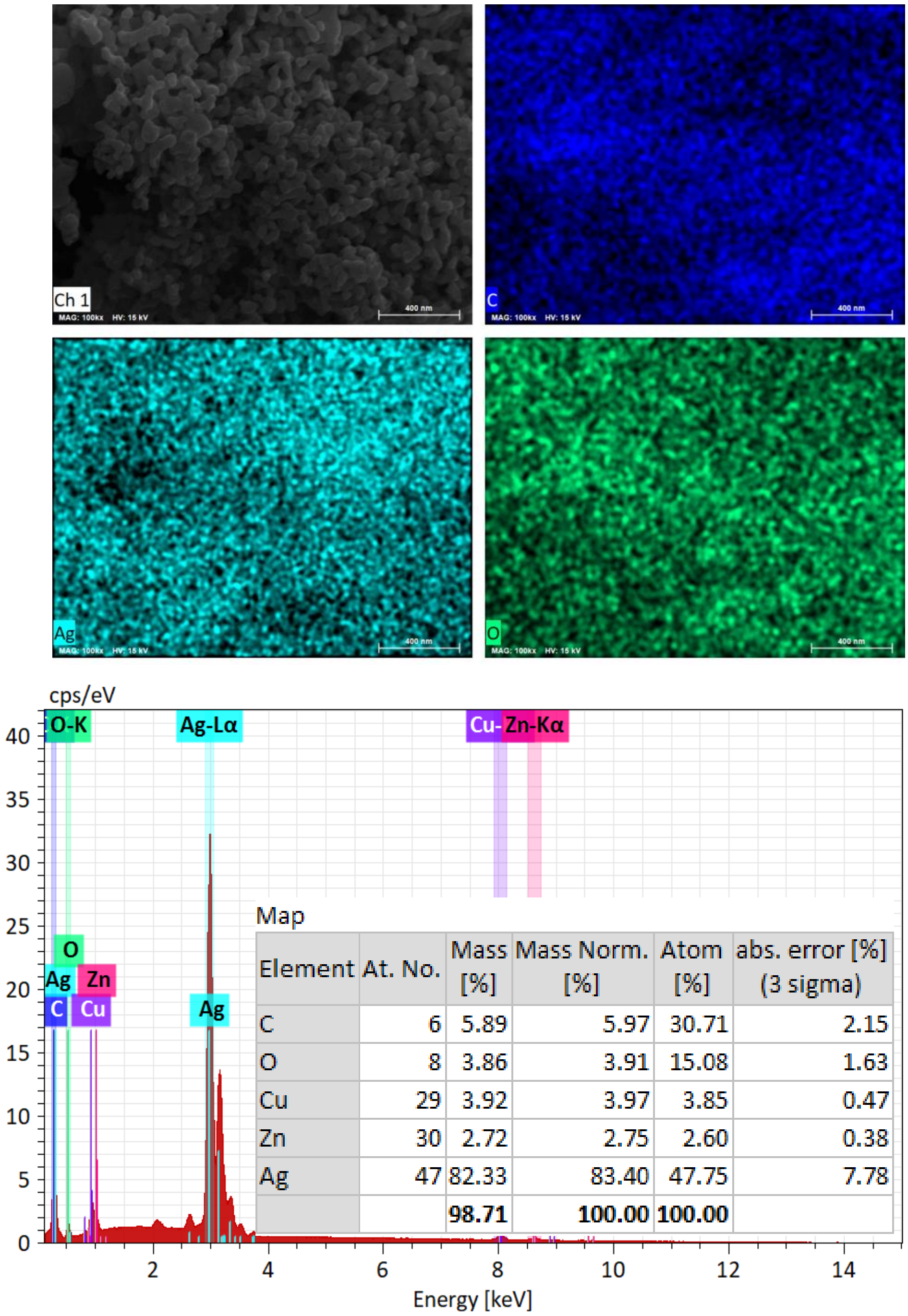

Map

Element	At. No.	Mass [%]	Mass Norm. [%]	Atom [%]	abs. error [%] (3 sigma)
C	6	5.89	5.97	30.71	2.15
O	8	3.86	3.91	15.08	1.63
Cu	29	3.92	3.97	3.85	0.47
Zn	30	2.72	2.75	2.60	0.38
Ag	47	82.33	83.40	47.75	7.78
		**98.71**	**100.00**	**100.00**	

Figure S2. EDX spectra of SNC-PC-X1 (Scale = 400 nm).

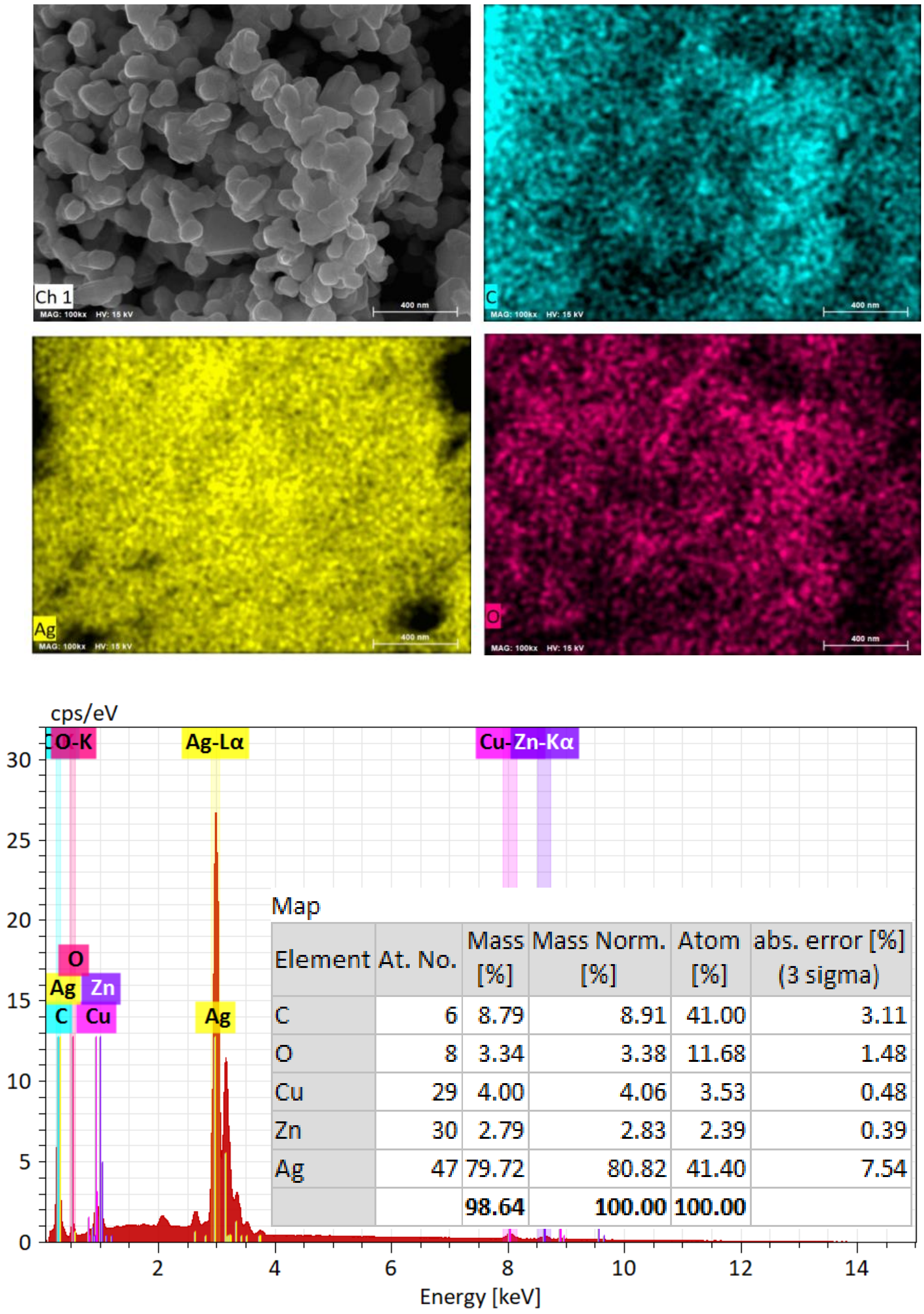

Element	At. No.	Mass [%]	Mass Norm. [%]	Atom [%]	abs. error [%] (3 sigma)
C	6	8.79	8.91	41.00	3.11
O	8	3.34	3.38	11.68	1.48
Cu	29	4.00	4.06	3.53	0.48
Zn	30	2.79	2.83	2.39	0.39
Ag	47	79.72	80.82	41.40	7.54
		**98.64**	**100.00**	**100.00**	

Figure 3. EDX spectra of SNC-PC-X2 (Scale = 400 nm).

X-ray Photoelectron Spectroscopy

The XPS technique is adopted to investigate the chemical and electronic structure at the molecule–metal interface and stability in capped nanoparticles samples [1]. X-ray photoemission measurements were carried out on SNC-PC-X1 and SNC-PC-X2 nanocomposites; for comparison, the model system SNP (Ref) was also investigated.

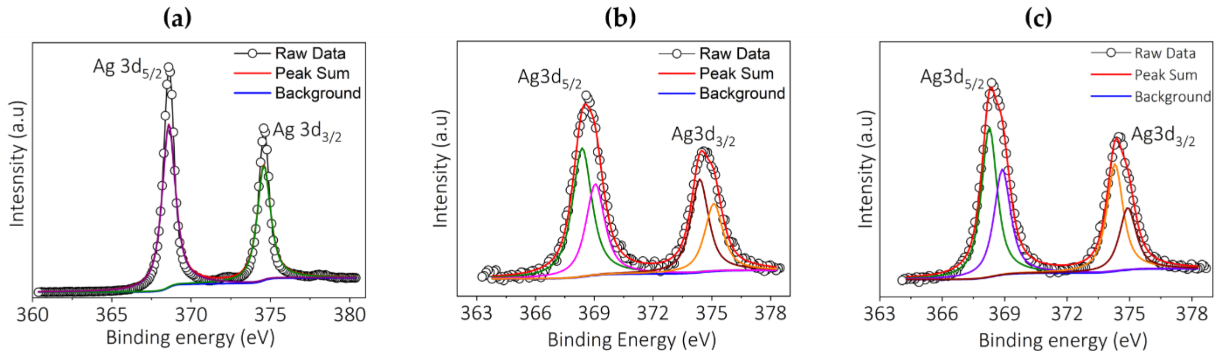

Figure S4. XPS measurements on SNPs (**a**) Ag3d spectra for SNP-Ref, (**b**) Ag3d spectra for SNC-PC-X1, and (**c**) Ag3d spectra for SNC-PC-X2.

Looking at the Ag3d core level spectra of SNC-PC-X1 and SNC-PC-X2 confirms the effective bonding between PC and SNPs. As shown in the Figure S4 (b & c), a pronounced shoulder in the pure metal Ag3d peak can be observed, attributing that silver atomic surface are covalently bonded to functional groups in PC. The Ag3d$_{5/2}$ component at about 368.2 eV corresponds to unperturbed metallic silver. The lower intensity peak contributions at higher BE are assigned to positively charged Ag atoms at the PC surface, chemically bonded to the pyridine groups. The two Ag3d spin-orbit pairs are separated of about 0.5-0.6 eV. Whereas no shoulder peak is observed in case of SNP (Ref), confirms that no bonding involved and absence of PC (Figure S4a) [2, 3].

References

1. Porcaro, F.; Battocchio, C.; Antoccia, A.; Fratoddi, I.; Venditti, I.; Fracassi, A.; Luisetto, I.; Russo, M.V.; Polzonetti, G. Synthesis of functionalized gold nanoparticles capped with 3-mercapto-1-propansulfonate and 1-thioglucose mixed thiols and “in vitro” bioresponse. *Colloids Surf. B Biointerfaces* **2016**, *142*, 408–416. doi: 10.1016/j.colsurfb.2016.03.016.
2. Porcaro, F.; Carlini, L.; Ugolini, A.; Visaggio, D.; Visca, P.; Fratoddi, I.; Venditti, I.; Meneghini, C.; Simonelli, L.; Marini, C.; Olszewski, W.; Ramanan, N.; Luisetto, I.; Battocchio, C. Synthesis and Structural Characterization of Silver Nanoparticles Stabilized with 3-Mercapto-1-Propansulfonate and 1-Thioglucose Mixed Thiols for Antibacterial Applications. *Materials* **2016**, *9*, 1028; doi:10.3390/ma9121028.
3. Cheng. X.; Liu, M.; Zhang, A.; Hu, S.; Song, C.; Zhang, G.; Guo, X. Size-controlled silver nanoparticles stabilized on thiol-functionalized MIL-53(Al) frameworks. *Nanoscale* **2015**, *7*, 9738–9745. doi: 10.1039/c5nr01292a.

© 2020 by the authors. Licensee MDPI, Basel, Switzerland. This article is an open access article distributed under the terms and conditions of the Creative Commons Attribution (CC BY) license (http://creativecommons.org/licenses/by/4.0/).
